# RNA methyltransferase NSUN2 promotes gastric cancer cell proliferation by repressing p57^Kip2^ by an m^5^C-dependent manner

**DOI:** 10.1038/s41419-020-2487-z

**Published:** 2020-04-24

**Authors:** Lin Mei, Cheng Shen, Ran Miao, Jing-Zi Wang, Mend-Da Cao, Yi-Sheng Zhang, Liang-Hui Shi, Guo-Hai Zhao, Ming-Hai Wang, Li-Sheng Wu, Ji-Fu Wei

**Affiliations:** 1 Department of General Surgery, The First Affiliated Yijishan Hospital of Wannan Medical College, 241001 Wuhu, China; 2 Department of General Surgery, The First Affiliated Hospital of University of Science and Technology of China, 230001 Hefei, China; 3 Research Division of Clinical Pharmacology, The First Affiliated Hospital with Nanjing Medical University, 300 Guangzhou Road, 210029 Nanjing, China

**Keywords:** RNA, Growth factor signalling

## Abstract

The RNA methyltransferase NSUN2 has been involved in the cell proliferation and senescence, and is upregulated in various types of cancers. However, the role and potential mechanism of NSUN2 in gastric cancer remains to be determined. Our study showed that NSUN2 was significantly upregulated in gastric cancers, compared to adjacent normal gastric tissues. Moreover, NSUN2 could promote gastric cancer cell proliferation both in vitro and in vivo. Further study demonstrated that CDKN1C (p57^Kip2^) was the potential downstream gene of regulated by NSUN2 in gastric cancer. NSUN2 could promote gastric cancer cell proliferation through repressing p57^Kip2^ in an m^5^C-dependent manner. Our findings suggested that NSUN2 acted as an oncogene through promoting gastric cancer development by repressing p57^Kip2^ in an m^5^C-dependent manner, which may provide a novel therapeutic target against gastric cancer.

## Introduction

During the past a few years, RNA modifications have been found to play an important role in the occurrence and development of many tumors. More than 100 types of chemical modifications have been identified in various types of RNAs, with methylation being the most common^1^. Methylation is a prevalent post-transcriptional modification that occurs in almost all RNA species. N^6^-methyladenosine (m^6^A) is the most abundant internal modification in mammalian messenger RNA (mRNAs) and widely involved in various biological processes of mRNAs^2–4^. Recently, many studies revealed that aberrant m^6^A modification is closely related to tumorigenesis, including acute myeloid leukemia^5^, hepatocellular carcinoma^6,7^, breast cancer^8,9^, bladder cancer^10,11^, cervical cancer^12^, and lung cancer^13^.

Another important RNA modification, 5-methylcytosine(m^5^C), was first identified in stable and highly abundant transfer RNAs (tRNAs) and ribosome RNAs (rRNAs)^14^. Recently, m^5^C modification and related m^5^C sites have been found in mRNA by advanced high-throughput techniques combined with next-generation sequencing in mRNAs. Yang et al.^15^ found that NSUN2 (NOP2/Sun domain family, member 2; MYC-induced SUN domain–containing protein, Misu) was the main enzyme catalyzing m^5^C formation, while Aly/REF export factor (ALYREF, an mRNA transport adaptor, also named THOC4) functioned as a specific mRNA m^5^C-binding protein regulating mRNA export. It was found that m^5^C could promote the pathogenesis of bladder cancer through stabilizing mRNAs^16^. Recent studies showed that NSUN2 was linked to cell proliferation, stem cell differentiation and testis differentiation^17,18^. Wang and colleagues^19^ found that NSUN2 could delay the replicative senescence by repressing Cyclin-dependent kinase inhibitor 1B (CDKN1B, p27^Kip1^) translation and promote cell proliferation by elevating Cyclin-dependent kinase 1 (CDK1) translation^20^. Moreover, elevated protein expression of NSUN2 was found in various types cancers, including the esophageal, stomach, liver, pancreas, uterine cervix, prostate, kidney, bladder, thyroid, and breast cancers by immunohistochemistry (IHC) analysis^21^. Indeed, Wang and colleagues^22^ found that NSUN2 was associated with metastatic progression by affecting DNA hypomethylation in human breast cancer. Gao et al.^23^ also found NSUN2 could promote tumor progression via its interacting partner RPL6 in gallbladder carcinoma. However, the role and related mechanisms of NSUN2 in gastric cancer has not been investigated.

In the present study, we firstly showed that NSUN2 was significantly upregulated in gastric cancers, compared to adjacent normal gastric tissues. Moreover, NSUN2 could promote the gastric cancer cells proliferation both in vitro and in vivo. Further study demonstrated that p57^Kip2^ was the potential downstream gene regulated by NSUN2 in gastric cancer. Furthermore, NSUN2 could promote gastric cancer cell proliferation by repressing p57^Kip2^ in an m^5^C-dependent manner. This study suggested that NSUN2-mediated m^5^C methylation of p57^Kip2^ mRNA may serve as novel mechanism for gastric cancer development and progression.

## Results

### NSUN2 was upregulated in human gastric cancers compared to adjacent normal gastric tissues

**Fig. 1:**
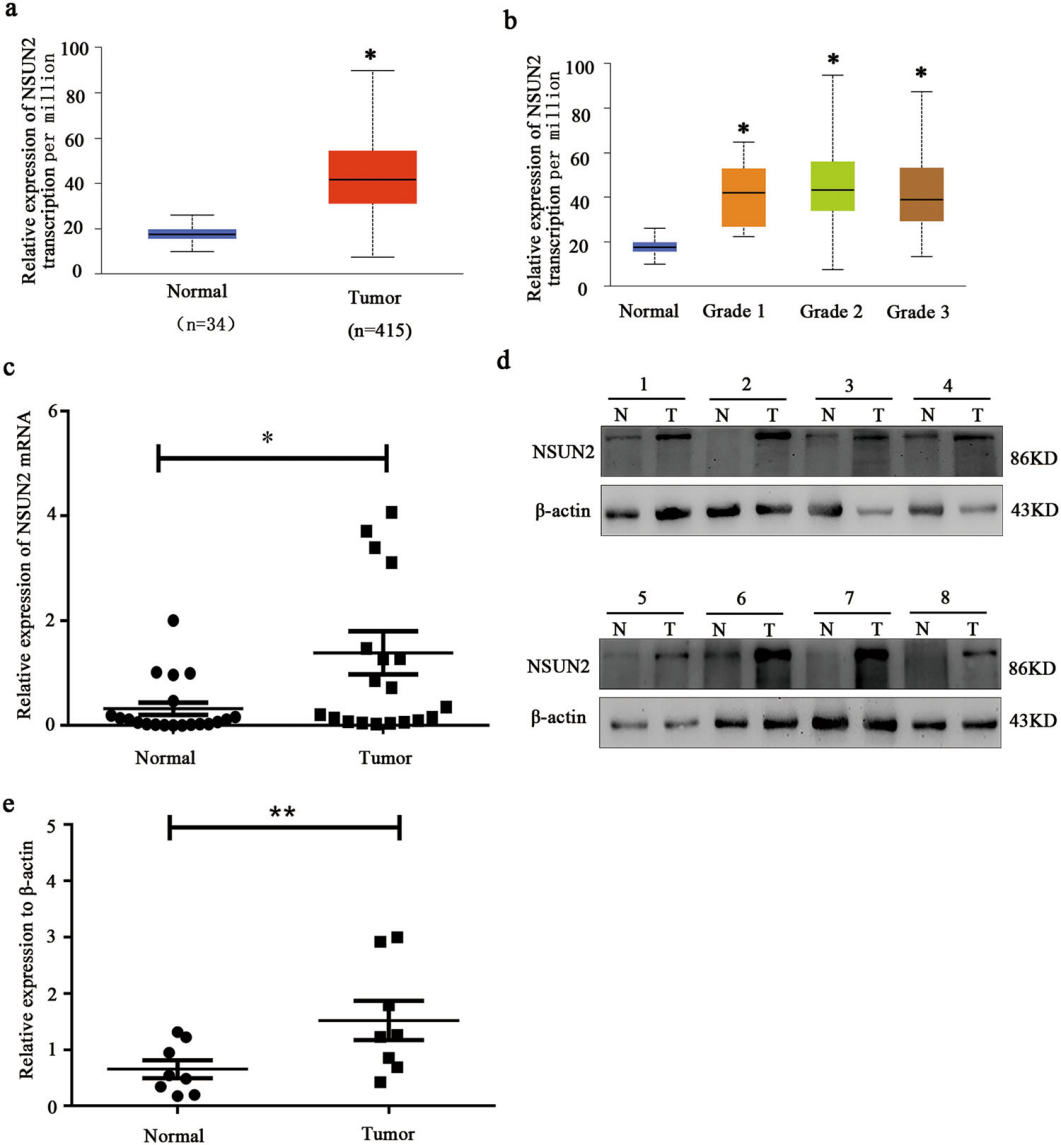
NSUN2 was upregulated in human gastric cancer tissues, compared to adjacent gastric normal tissues. **a**, **b** NSUN2 was significantly upregulated in gastric cancer tissues, compared with adjacent gastric normal tissues from the TCGA database, *p < 0.05; **c** Relative expression of NSUN2 mRNA in gastric cancer tissues and compared with corresponding adjacent normal gastric tissues. NSUN2 expression was examined using qRT-PCR and normalized to β-actin expression. The horizontal lines and numbers represent the median values of the distribution. *p < 0.05. **d** Expression of NSUN2 at protein level in eight paired gastric cancer tissues and adjacent normal gastric tissues by western blot. T: Gastric tumor tissues, N: Adjacent normal tissues. **e** The protein levels of NSUN2 were quantified by densitometry and the relative gray value of NSUN2 protein (normalized to β-actin) in gastric cancer tissues and adjacent gastric normal tissues was statistically significant. *p < 0.05.

Firstly, TCGA database analysis showed that NSUN2 was upregulated in tumors compared to adjacent normal gastric tissues (Fig. 1a, b). Meanwhile, to determine NSUN2 expression in gastric cancer tissues, we examined expression of NSUN2 in gastric cancer patients’ tissues by performing quantitative real-time PCR (qRT-PCR) and western blot assay. As shown in Fig. 1c–e, both the mRNA and protein expressions of NSUN2 was significantly upregulated in gastric tissues, compared to corresponding adjacent normal gastric tissues. These findings implied that NSUN2 was upregulated in human gastric cancer, compared to adjacent normal gastric tissues.

### NSUN2 promoted the human gastric cancer proliferation in vitro

**Fig. 2:**
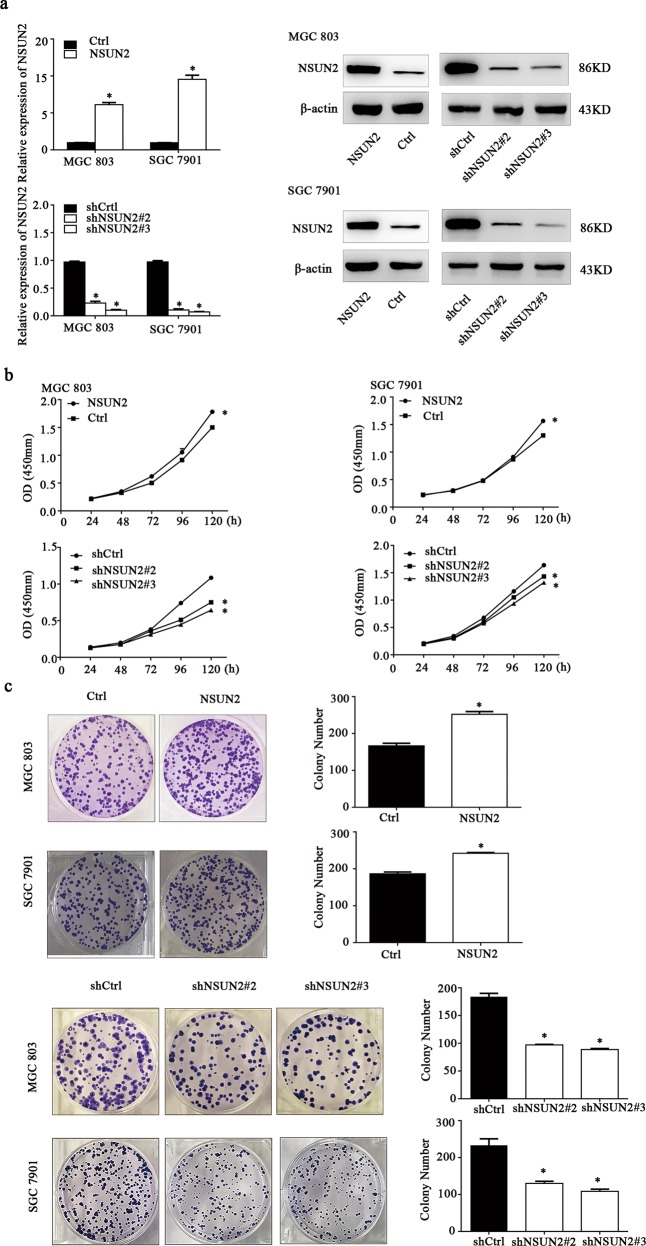
NSUN2 promoted the human gastric cancer proliferation in vitro. **a** qRT-PCR analysis of overexpress and knockdown of NSUN2 by lentiviral constructs in MGC 803 and SGC 7901 cells. NSUN2, pLOV-NSUN2; Ctrl, pLOV-control vector shNSUN2#2, pLKD-shNSUN2-1; shNSUN2#3, pLKD-shNSUN2-2; shCtrl, pLKD-control vector. Data were presented as the mean ± SD; *p < 0.05. **b**, **c** CCK-8 and colony formation assays were performed to determine the growth ability of overexpress and knockdown of NSUN2. Data were presented as the mean ± SD; *p < 0.05.

Firstly, qRT-PCR and western blot assay revealed that NSUN2 was stably knockdown or overexpression in SGC 7901 and MGC 803 cells (Fig. 2a). Subsequently, the CCK-8 assay and colony formation assay showed that NSUN2 knockdown significantly inhibited cell proliferation and colony formation, whereas NSUN2 overexpression promoted cell proliferation and colony formation (Fig. 2b, c). Flow cytometry analysis further revealed that NSUN2 knockdown induced at the G1/G0 cell cycle arrest and NSUN2 overexpression decreased the percentage of G1/G0 phase, compared with wild-type cells, both in SGC 7901 and MGC 803 cells (Additional file: Supplementary Fig. S1a, b). Taken together, our results suggested that NSUN2 could promote the gastric cancer cell proliferation in vitro.

### NSUN2 promoted human gastric cancer tumorigenesis in vivo

**Fig. 3:**
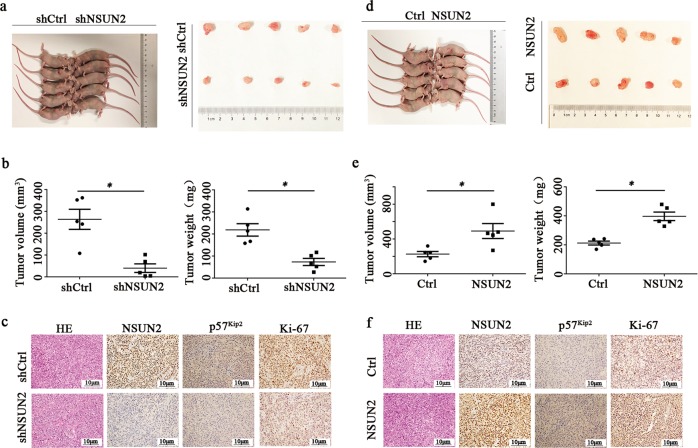
NSUN2 promoted human gastric cancer tumorigenesis in vivo. NSUN2 overexpress cells, NSUN2 knockdown cells and corresponding wild-type cells were injected into female nude mice subcutaneously. **a**, **d** Tumor-bearing mice and tumors were shown. Data were showed as the mean ± SD; *p < 0.05. **b**, **e** Tumor size and weight were measured after 3 weeks. Data were showed as the mean ± SD; *p < 0.05**. c**, **f** HE and IHC staining of NSUN2, p57^Kip2^, and Ki-67 expression in tumor sections at ×200 magnification. Scale bars indicated 10 μm.

MGC 803 cell line was used in this experiment. Firstly, stable NSUN2 overexpress cells, NSUN2 knockdown cells and their corresponding wild-type cells were injected into female nude mice. Up to 3 weeks after injection, we found that stable knockdown of NSUN2 suppressed tumor growth in nude mice effectively. The average tumor weight and volume of NSUN2 knockdown cells group were also significantly lower than those in the wild-type group (Fig. 3a, b). Moreover, IHC analysis confirmed that the tumors formed from the wild-type cells group displayed stronger Ki-67 staining than those from NSUN2 knockdown cells group (Fig. 3c). whereas, overexpression of NSUN2 significantly promoted tumor growth in nude mice (Fig. 3d–f). Our results indicated that NSUN2 could promote human gastric cancer tumorigenesis in vivo.

### p57^KIP2^ was identified as potential target regulated by NSUN2 by RNA sequencing (RNA-seq)

To identify the downstream targets regulated by NSUN2 in gastric cancer, we performed RNA-Seq assay to determine the mRNA expression changes in NSUN2 knockdown and corresponding wild-type cells. In this study, Kyoto Encyclopedia of Genes and Genome (KEGG) analysis revealed that differentially expressed genes were significantly changed in ribosome, endocytosis, cell cycle, protein processing in endoplasmic reticulum and apoptosis, suggesting that NSUN2 may play important roles in protein modification and cell proliferation (Table 1). As cell proliferation plays an important role in tumor development, we selected cell cycle related pathways as a candidate targets of NSUN2 for further study. In total, we found 86 differential genes expression from cell cycle related pathways in NSUN2 stable knockdown cells, compare with corresponding wild-type cells (Table 2). We then found that p57^Kip2^ was obviously upregulated among these genes and was identified as potential target regulated by NSUN2.

**Table 1:** Enrichment list of differential gene in KEGG, which indicated that cell cycle pathways were significantly associated with NSUN2.

Pathway	Input number	Background number	*p*-value
Ribosome	118	138	*p* < 0.05
Endocytosis	171	260	*p* < 0.05
**Cell cycle**	**86**	**124**	***p*** < **0.05**
Protein processing in endoplasmic reticulum	110	166	*p* < 0.05
Apoptosis	95	140	*p* < 0.05
Small cell lung cancer	62	86	*p* < 0.05
Non-alcoholic fatty liver disease (NAFLD)	97	151	*p* < 0.05
Lysosome	81	123	*p* < 0.05
Prostate cancer	61	89	*p* < 0.05
p53 signaling pathway	49	69	*p* < 0.05

The significance of row given in bold in the table is to highlight the direction of research.

**Table 2:** Ten of 86 differential genes enriched in cell cycle pathway of RNA-Seq.

Gene names	Read count -shNSUN2	Read count -shCtrl	Log^2^ fold change	*p*-value
*GADD45B*	237.5057421	2772.463549	–3.5451	*p* < 0.05
*GADD45A*	576.4076242	4696.206607	–3.0263	*p* < 0.05
***CDKN1C***	**3425.9955**	**874.8185493**	**1.9695**	***p*** < **0.05**
*BUB1*	1811.984441	579.1016155	1.6457	*p* < 0.05
*MYC*	4413.944716	13606.89379	–1.6242	*p* < 0.05
*FZR1*	972.1006731	2877.720829	–1.5657	*p* < 0.05
*CCNH*	223.3793522	635.0115061	–1.5073	*p* < 0.05
*TP53*	909.4904515	2391.549555	–1.3948	*p* < 0.05
*CDKN1A*	4261.347275	11181.6708	–1.3918	*p* < 0.05
*SKP2*	2317.478004	921.422872	1.3306	*p* < 0.05

The significance of row given in bold in the table is to highlight the direction of research.

### NSUN2 destabilized the p57^Kip2^ transcript and it may be involved in the oncogenic function of NSUN2 in gastric cancer

**Fig. 4:**
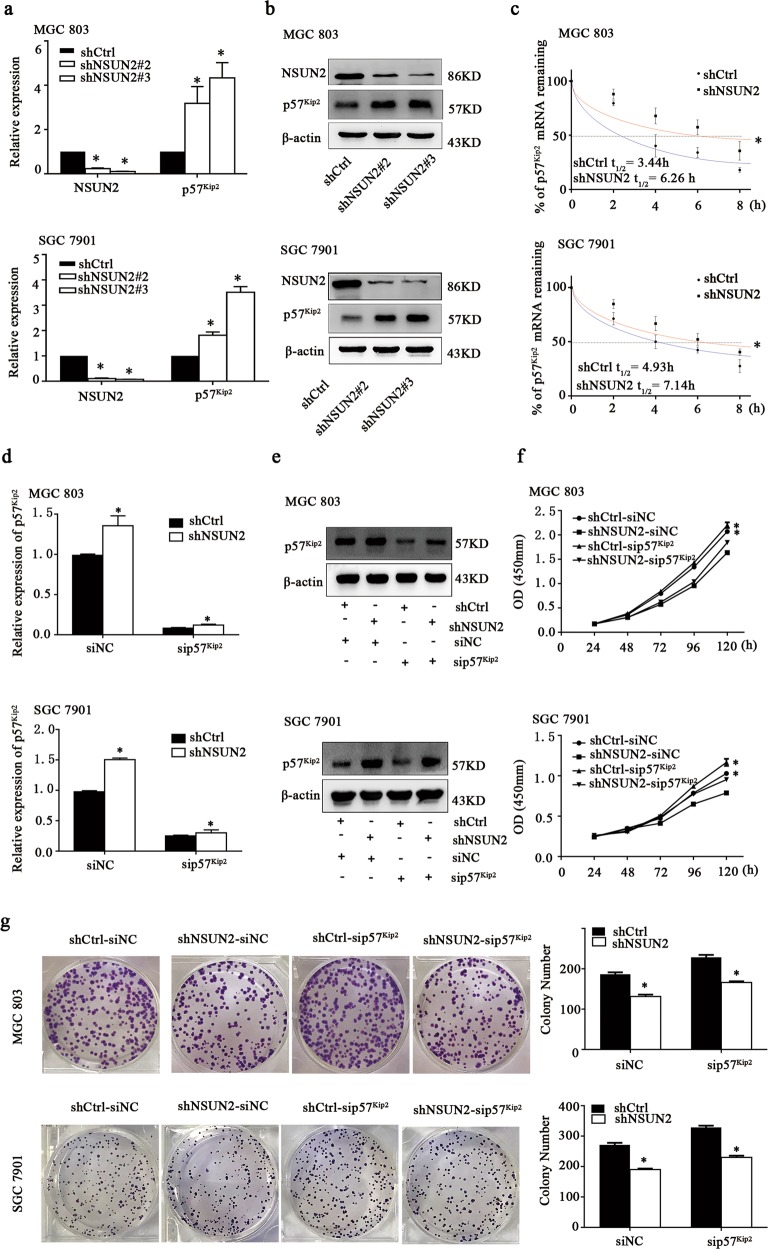
p57^KIP2^ was identified as potential target regulated by NSUN2 by RNA sequencing. **a** RNA was isolated from SGC 7901 and MGC 803 cells with stable knockdown of NSUN2 and p57^Kip2^ mRNAs expression was determined by qRT-PCR. Data were showed as the mean ± SD; *p < 0.05. **b** The protein levels of p57^Kip2^ and β-actin was assessed by western blot. **c** The mRNA half-life (t1/2) of p57Kip2 in SGC 7901 and MGC 803 cells with stable knockdown of NSUN2 or corresponding wild-type cells. **d**, **e** The relative mRNA and protein expression of p57^Kip2^ in NSUN2 knockdown cells or corresponding wild-type cells, transfected with small-interfering RNAs (siRNAs), was tested using qRT-PCR and western blot. siNC: negative control siRNA; sip57^Kip2^: siRNA against p57^Kip2^. Data were showed as the mean ± SD; *p < 0.05. **f**, **g** CCK-8 and colony formation assays were performed to determine the growth ability of NSUN2 knockdown cells after transfection of siRNA against p57^Kip2^. Data were showed as the mean ± SD; *p < 0.05.

The level of p57^Kip2^ was remarkably upregulated in NSUN2 knockdown cells identified by qRT-PCR and Western blot (Fig. 4a, b). In addition, we found the relative half-life of p57^Kip2^ mRNA increased from 3.44 to 6.26 h in MGC 803 cells, and from 4.93 to 7.14 h in SGC 7901 cells, following the NSUN2 knockdown (Fig. 4c). We silenced p57^Kip2^ via transfecting with siRNA in NSUN2 knockdown cells and wild-type cells (Fig. 4d, e). Upon silencing of p57^Kip2^ expression by siRNAs, the ability of cell proliferation in both NSUN2 knockdown cells and wild-type cells was enhanced (Fig. 4f, g). These results suggested that NSUN2 might exert its oncogenic effects in gastric cancer cells by repressing p57^Kip2^ expression.

### NSUN2 destabilized the p57^Kip2^ mRNA relies on its methyltransferase activity and m^5^C modifications in the 3′- untranslated region (UTR) of p57^Kip2^ mRNA

**Fig. 5:**
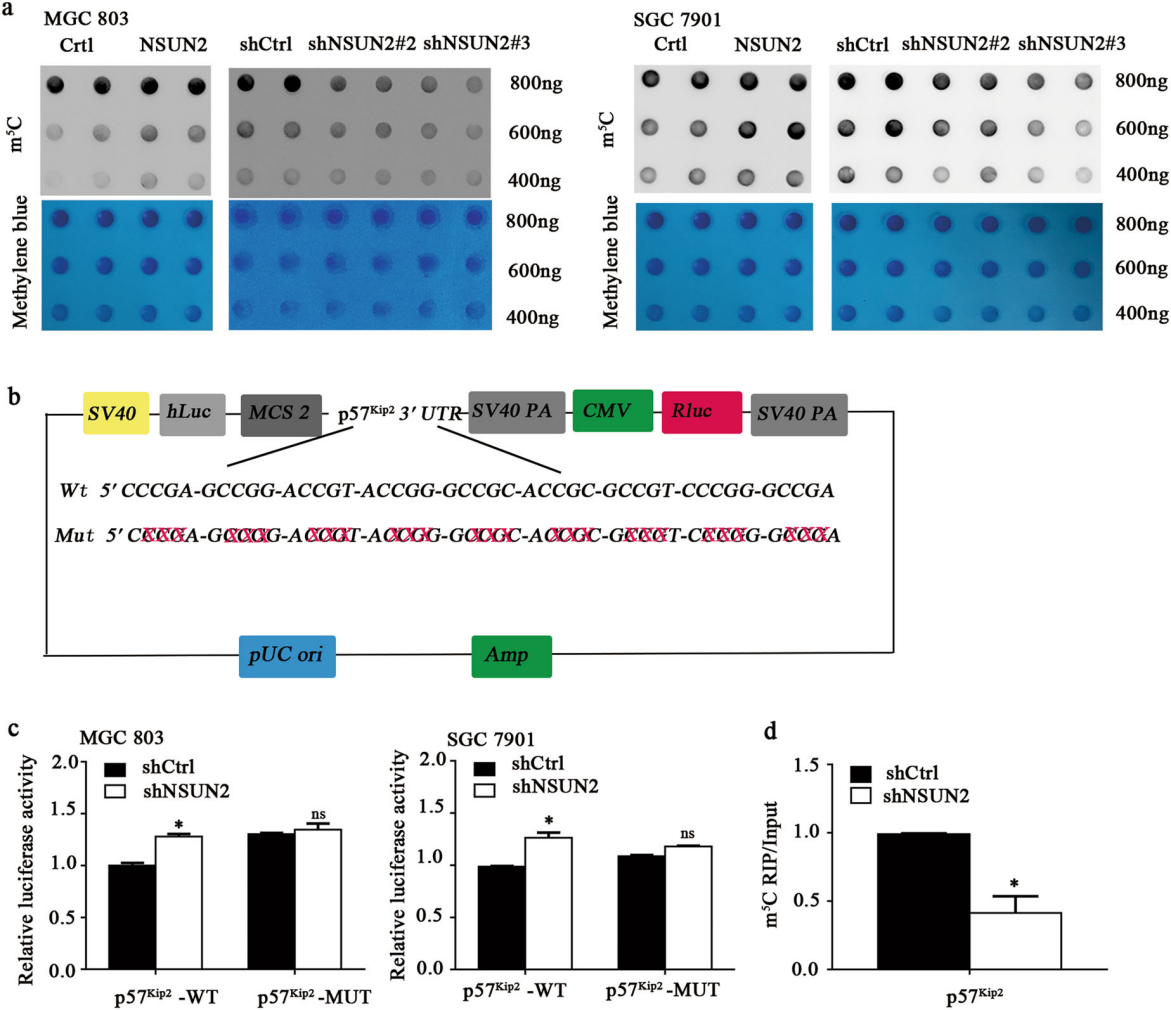
NSUN2 destabilized the p57^Kip2^ mRNA relies on its methyltransferase activity and m^5^C modifications in the 3′- UTR of p57^Kip2^ mRNA. **a** m^5^C dot blot assay of overexpress or knockdown of NSUN2 in MGC 803 and SGC 7901 cells, methylene blue staining (as control). **b**, **c** Pezx-FR02- p57^Kip2^ -3′-UTR plasmid with either wild-type or mutant (CCT mutation) m^5^C sites were constructed. The pattern diagram was shown. Above constructed plasmid was transfected into stable knockdown of NSUN2 or corresponding wild-type cells. Firefly luciferase activity was measured and normalized to Renilla luciferase activity. Data were presented as the mean ± SD; *p < 0.05. **d** m^5^C RIP and qRT-PCR analysis of m^5^C level in mRNA of p57^Kip2^ in MGC 803 cells transduced with stable knockdown of NSUN2 or corresponding wild-type cells. Data were showed as the mean ± SD; *p < 0.05.

The Dot blot assay showed that NSUN2 knockdown significantly decreased the m5C levels, whereas NSUN2 overexpression increased the m^5^C levels (Fig. 5a). To assess the role of the m^5^C modifications in p57^Kip2^ mRNA regulated by NSUN2, we conducted wild-type and mutant 3′-UTR of p57^Kip2^ reporter plasmids for the luciferase reporter assays (Fig. 5b). Relative luciferase activity of the wild-type and mutant 3′-UTR of p57^Kip2^ reporter genes was measured in MGC 803 and SGC 7901 cells. As expected, the luciferase activity of the wild-type 3′-UTR of p57^Kip2^ reporter gene was significantly enhanced after NSUN2 silencing. However, knockdown of NSUN2 had no effect on the expression of the mutated 3′-UTR of p57^Kip2^ reporter gene (Fig. 5c). More importantly, by m^5^C RNA immunoprecipitation (RIP) assay and qPCR assay, we found that m^5^C antibody significantly enriched 3′-UTR of p57^Kip2^ mRNA and knockdown of NSUN2 reduced the m^5^C levels on 3′-UTR of p57^Kip2^ mRNA (Fig. 5d). Altogether, our data indicated that NSUN2 destabilized the p57^Kip2^ mRNA relies on its m^5^C methyltransferase activity in its 3′-UTR.

## Discussion

Gastric cancer is the most common gastrointestinal tumors, representing one of the leading causes of cancer-related deaths worldwide^24,25^. Despite the improvement in surgical techniques and patient management, there has been unsatisfactory improvement in the 5-year overall survival rate. Many patients were diagnosed with advanced stages that limited the successful therapeutic strategies. Furthermore, the molecular mechanisms underlying gastric cancer progression is still poorly understood. Therefore, better understanding of the tumor formation and diagnostic markers will improve the diagnosis and treatment of gastric cancer.

m^5^C modification is another important post-transcriptional RNAs modification beside m^6^A modification. As a main m^5^C methyltransferase, NSUN2 was reported to promote cell proliferation, mobility, invasion in breast cancer^22^, gallbladder carcinoma^23^, and associated with poor prognosis in head and neck squamous carcinoma by bioinformatics analysis^26^. However, NSUN2 was few studied in tumors formation related its m^5^C modification activity, especially gastrointestinal cancers. In this study, we found NSUN2 was upregulated in gastric cancer tissues, compared to adjacent normal gastric tissues in mRNA and protein levels. Interestingly, there is much more heterogeneity at protein than mRNA levels both in gastric cancer tissues and normal adjacent tissues. There may be multiple reasons for inconsistent changes in proteins and mRNAs^27,28^. Post-translational regulation of NSUN2 may be one of the important reasons, which may be different in various gastric cancer cases. Its detailed mechanism needs further investigation. Subsequently. We also found NSUN2 could increase gastric cancer cells proliferation both in vitro and in vivo significantly.

Subsequent RNA-seq and KEGG analysis found that cell cycle was the main pathway regulated by NSUN2 in gastric cancer. Cell cycle dysregulation is a hallmark of cancer due to uncontrolled proliferative signaling^29^. The affected transitions in the cell cycle are regulated by the balanced activities of cyclin-dependent kinases (CDKs) and CDK inhibitors. Although p57^Kip2^ might not be the only targeted gene of NSUN2, our results confirmed that p57^Kip2^ was an important downstream gene regulated by NSUN2 in gastric cancer. p57^Kip2^ is the recently found CDK inhibitors of the Cip/Kip family, and has been involved in many biological processes, including cell cycle control, differentiation, apoptosis, tumorigenesis and development^30,31^. Recent studies indicated that p57^Kip2^ was frequently downregulated in multiple types of human cancers such as breast cancer, hepatocellular carcinoma, colorectal cancer, and ovarian cancer^32,33^. Importantly, De and colleagues^34^ found that p57^Kip2^ could serve as a tumor suppressor in gastric cancer. In our study, we found that the expression level of NSUN2 was negatively correlated with p57^Kip2^ and the ability of NSUN2 knockdown cells proliferation was enhanced after p57^Kip2^ silencing in gastric cancer. It revealed another regulatory mechanism that NSUN2 play an oncogenic role by repressing p57^Kip2^ expression in gastric cancer.

Previous studies showed that RNA methylase play potential role in regulating mRNA decay, translation, and processing^35^. In our study, we demonstrated that relative half-life of p57^Kip2^ mRNA increased in NSUN2 knockdown cells. These results indicated that RNA methyltransferase NSUN2 could affect p57^Kip2^ mRNA stability. Wang et al. found that different functional mechanisms of RNA methyltransferase NSUN2 depended on the location of methylation by m^5^C modification^19,20,36–38^. Our dual-luciferase reporter assay also found that the luciferase activity of the wild-type 3′-UTR of p57^Kip2^ reporter gene was significantly enhanced, compared with the mutated 3′-UTR of p57^Kip2^ reporter gene. Subsequently, our m^5^C RIP and qRT-PCR assays found that m^5^C antibody significantly enriched 3′-UTR of p57^Kip2^ mRNA and knockdown of NSUN2 reduced the m^5^C levels on 3′-UTR of p57^Kip2^ mRNA, indicating that RNA methyltransferase NSUN2 regulated p57^Kip2^ expression by m^5^C modification in 3′-UTR of its mRNA. Based on the above results, we elaborated a novel mechanism indicating that NSUN2 mainly methylated the 3′-UTR of p57^Kip2^ mRNA, which led to the downregulation of p57^Kip2^ at the RNA level and related protein levels. In the present study, we explored the effects of NSUN2 on gastric cancer cell cycle progression and demonstrated that NSUN2 could repress p57^Kip2^ by an m^5^C-dependent manner.

In summary, we found that NSUN2 acted as an oncogene through promoting gastric cancer development by repressing p57^Kip2^ in an m^5^C-dependent manner, which may provide a novel therapeutic target against gastric cancer.

## Materials and methods

### Bioinformatics analysis

Clinical data for Bioinformatics analysis were downloaded from The Cancer Genome Atlas (TCGA) database (https://cancergenome.nih.gov/), and applied for analyzing the expression of NSUN2 in 415 gastric cancer tissues and 34 normal tissues.

### Clinical sample

Twenty pairs of gastric tumor and adjacent normal tissues were obtained from the First Affiliated Yijishan Hospital with Wannan Medical College from 2017 to 2018. All samples were obtained with written informed consent from patients and the ethics committee of Wannan Medical College approved these tissues for research use.

### Cell culture and transfection

Human gastric cancer cell lines MGC 803 and SGC 7901 were obtained from the Chinese Academy of Sciences Committee on Type Culture Collection Cell Bank (shanghai, China), and has recently been tested for mycoplasma contamination. The cells were maintained in RPMI 1640 medium (GIBCO, USA) supplemented with 10% fetal bovine serum (GIBCO, USA), 100 U/ml penicillin–streptomycin (GIBCO, USA). All the cell lines were maintained at 37 °C, 5% CO_2_.

Lentivirus constructs for NSUN2 overexpression and knockdown were obtained from Obio Technology (Shanghai, China), and generated as described previously^39^. Briefly, the gastric cancer cells were stably transfected with NSUN2 overexpression lentivirus (termed as NSUN2) and negative control (termed as Ctrl) using polybrene (Obio Technology, China). Similarly, cells were stably transfected with negative control (termed as shCtrl) and NSUN2 knockdown lentivirus (termed as shNSUN2#2 and #3). Subsequently, stably transfected gastric cancer cells were used for further studies by selection using puromycin (5 μg/ml) for 1–2 weeks.

To explore the further relationship between NSUN2 and p57^Kip2^ in gastric cancer cells, small-interfering RNAs (siRNAs) against p57^Kip2^ or negative control RNAs were purchased from GenePharma (Shanghai, China), and transfected into the stably transfected gastric cancer cells using EndoFectin^TM^-Max (GeneCopoeia, China) according to the manufacturer’s instructions.

### RNA isolation, reverse transcription, and qRT-PCR

Total RNA was isolated from tissues and cell lines using Trizol reagent (Invitrogen, USA) according to the manufacturer’s protocol. cDNA was synthesized by reverse transcription by 1 or 2 μg RNA using Primescript^TM^reverse transcription reagent (Takara, Japan). qRT-PCR was performed with SYBR Green PCR Kit (Takara, Japan) in the StepOnePlus system (Applied Biosystems, USA). Primer pairs used were as follows: NSUN2, forward primer: 5′-GAACTTGCCTGGCACACAAAT-3′, reverse primer: 5′-TGCTAACAGCTTCTTGACGACTA-3′.

p57^Kip2^, forward primer: 5′-GCGGCGATCAAGAAGCTGT-3′, reverse primer: 5′-GCTTGGCGAAGAAATCGGAGA-3′.

β-actin, forward primer: 5′-GAACTTGCCTGGCACACAAAT-3′, reverse primer: 5′-TGCTAACAGCTTCTTGACGACTA-3′. The fold change of gene expression was presented by the 2^–ΔΔCt^ method and normalize base on β-actin.

### Western blot

Cells were lysed completely in RIPA lysis buffer supplemented with 1% phenylmethanesulfonyl fluoride (PMSF) and 0.1% protease inhibitor cocktail (Beyotime, China) at 4 °C. The protein concentrations were determined using a bicinchoninic acid (BCA) protein assay kit (Beyotime, China). Total protein lysates were separated by sodium dodecyl sulfate–polyacrylamide gel electrophoresis and transferred to polyvinylidene fluoride membranes (PVDF, Millipore, USA). The membranes were incubated with primary antibodies and then with specific secondary antibodies after washed three times with 0.1% Tris-HCl with Tween-20 (TBST). The membranes were probed using immobilon Western chemiluminescent horseradish peroxidase substrate (Millipore, USA) and autoradiographed (TANON 5200, China). β-actin antibody was used as internal control. anti-NSUN2 (3H24L11) antibody was provided by ThermoFisher Scientific (USA). anti-p57^Kip2^ (ab75974) antibody was purchased from Abcam (USA). β-actin (8H10D10), anti-rabbit (#7074), and anti-mouse (#7076) secondary antibodies were obtained from Cell Signaling technology (USA).

### RNA m^5^C dot blot assay

Total RNA was first isolated from stable NSUN2 overexpression and knockdown cells and their corresponding negative control cells, and then treated with deoxyribonuclease I (DNase) according to the manufacturer’s protocol. RNA quality was analyzed by NanoDrop 2000 (Thermo Scientific, USA). Different amounts of RNA (400,600,800 ng) were loaded onto the Amersham Hybond N^+^ membrane (GE Healthcare) fixed on the Bio-Dot apparatus (Bio-Rad). After ultraviolet rays crosslinking for 5 min at 254 nm, the membrane was blocked with 5% non-fat dried milk in phosphate buffer solution with Tween-20 (PBST) followed by incubation with the primary mouse anti-m^5^C antibody (ab10805, Abcam, USA) and corresponding anti-mouse secondary antibody. After, the membranes were washed three times with 0.1% PBST, the intensity of the dot blot was determined by autoradiographed and analyzed by image J.

### Cell proliferation assay

The transfected gastric cells were monitored in 96 wells at ~2000 cells per well incubated at 37 °C in an atmosphere of humidified air 5% CO_2_ incubator, and the cell proliferation assay was carried out with cell counting kit-8 (CCK-8, Obio Technology, China) following the manufacturer’s protocol after 5 days of culture. Optical densities (ODs) were measured at 490 nm with a microplate reader (BioTek, USA). For colony formation assay, a certain number of cells were placed into each well of 6-well plates and cultured for 14 days before stained 0.5% crystal violet (Beyotime, China). The colonies with more than 50 cells was manually counted.

### Flow cytometry analysis

The transfected gastric cells were seeded in 96-well plates and harvested by trypsinization with 0.25% EDTA (Sigma, USA), when the cells were grown to 80% confluence. Cells for cell cycle were stained with propidium iodide (BD Biosciences, USA) and the percentages of cells in G0/G1, S, and G2/M phase were analyzed by flow cytometry (CtytoFLEX, Beckman,USA) and ModFit LT 5.0software.

### Tumor xenograft

Four- to eight-week-old female BALB/c nude mice were purchased from model animal research center of Nanjing University, and randomly divided into four groups (five per group). Five mice/group were subjected to the experiment to have statistical importance. Sable NSUN2 overexpression cells, NSUN2 knockdown cells and their corresponding wild-type cells (6 × 10^6^ cells in 100 ml PBS) were injected into the upper left flank region of each nude mice. Mice were sacrificed and tumor tissues were collected after 3 weeks. Tumor volumes (1/2 × length × width^2^) and weight were measured in mice. Hematoxylin-eosin (HE) staining and IHC were performed on processed and sectioned tissues from Serviebio company (Wuhan, China). The investigators were not blinded to the mice group during experiments. All procedures were approved by the animal care and use committee of Nanjing Medical University (acceptance no.: IACUC1804027).

### RNA-seq

RNA samples were isolated from stable NSUN2 knockdown cells and corresponding negative control cells in MGC 803 cells. The RNA samples were sequenced by the Allwegene Technology Inc (Beijing, China). Three biological replicates for each sample were included in this experiment. The threshold for screening differential genes (DEG) is generally as follows: |log2(Foldchange)| > 1, *q*-value < 0.05 and read count ≥100. DEG function analysis was by Gene Ontology (GO) and KEGG.

### RNA stability assay

To analyze RNA stability, stable NSUN2 knockdown cells and corresponding wild-type cells were treated with actinomycin D (1 μg/ml). Cells were collected at different time points (0, 2, 4, 6, 8 h), and RNA was extracted using Trizol reagent. Reverse transcription was performed using oligo primers and mRNA levels were measured using qRT-PCR.

### Dual-luciferase reporter assay

Dual-luciferase reporter assay was performed using Luc-Pair^TM^ Duo-luciferase HS assay kit (GeneCopoeia, USA) according to the manufacturer’s instructions. Wild-type and mutant 3′-UTR of p57^Kip2^ reporter plasmid were constructed from GenePharma (USA). Briefly, wild-type 3′-UTR of p57^Kip2^ reporter plasmid was made by inserting the 3′-UTR of p57^Kip2^ transcript after the Fluc coding sequence, and mutant 3′-UTR of p57^Kip2^ reporter plasmid was made by inserting the 3′-UTR of p57^Kip2^ transcript without “CCG” sequence according to Yang et al.^15^. Stable NSUN2 knockdown and corresponding negative control cells were plated in 96 wells dishes and infected with 100 ng of wild-type and mutated 3′-UTR of p57^Kip2^ transcript reporter plasmid using EndoFectin^TM^-Max (GeneCopoeia, China). After 24 h, cells were collected and assayed with NanoDrop 2000 (Thermo Scientific, USA). Firefly luciferase (F-luc) was used to assess the effect of m^5^C modification on p57^Kip2^ expression. Renilla Luciferase (R-luc) was used to standardize the transfection efficiency of the reporter plasmid.

### m5C RNA RIP assay

For m^5^C RIP, the standard procedure was described as previous study with some modifications^37,38^. Briefly, total RNAs were firstly isolated and treated with DNase. Then, total RNAs were chemically fragmented (~100 nucleotide) with 1×fragmentation buffer (100 mM Tris-HCl, 100 mM ZnCl_2_) and incubated with the m^5^C antibody (Abcam, USA) in IP buffer (50 mM Tris-HCl, 750 mM NaCl and 0.5% (vol/vol) Igepal CA-630). The IP samples were washed with elution buffer [1×IP buffer, 6.7 mM 5-methylcytosine hydrochloride (Sigma, USA)]. Enrichment of m^5^C containing mRNA was analyzed by RT-PCR.

### Statistical analysis

All experiments in this study were repeated in triplicate, unless otherwise specified. All dates were presented as the mean ± SD, and student’s *t*-tests (unpaired, two-tailed) were performed using the SPSS 19.0 software (SPSS, Chicago, IL, USA) and graphical presentations were conducted with GraphPad Prism 7.0 software (San Diego, CA). *p-*value < 0.05 was statistically significant.

## Supplementary information

Supplemental Figure Legend

Figure S1
