# Supplementary material for: RNA methyltransferase NSUN2 promotes gastric cancer cell proliferation by repressing p57Kip2 by an m5C-dependent manner
Source: Cell Death Dis. 2020 Apr 24;11(4):270. doi: 10.1038/s41419-020-2487-z (PMC7181747; doi:10.1038/s41419-020-2487-z)
Supplement: Supplementary file 1 — Supplemental Figure Legend [file 41419_2020_2487_MOESM1_ESM.docx]

**Supplemental Figure Legend**

**Figure S1: Knockdown of NSUN2 induces G1/S transition arrest**

**a-b,** Cell cycle of overexpress or knockdown of NSUN2 in MGC 803 and SGC 7901 cells were analyzed by flow cytometry. Representative profiles were shown on the left and the percentage of cells was statistically analyzed on the right. Data were showed as the mean ± SD; **p* <0.05.
