# Supplementary figures and images for: RNA methyltransferase NSUN2 promotes gastric cancer cell proliferation by repressing p57Kip2 by an m5C-dependent manner
Source: Cell Death Dis. 2020 Apr 24;11(4):270. doi: 10.1038/s41419-020-2487-z (PMC7181747; doi:10.1038/s41419-020-2487-z)

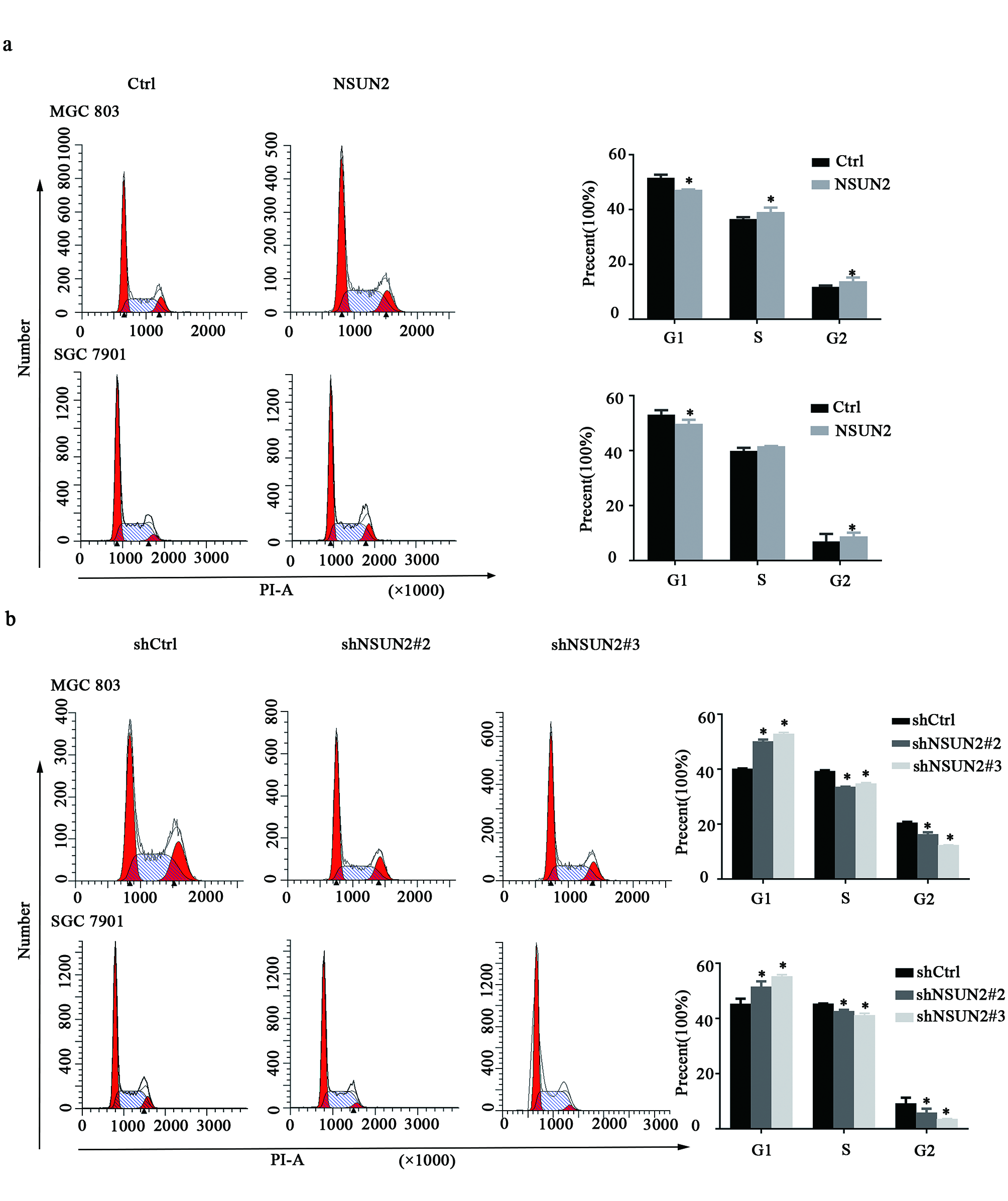

Supplement: Supplementary file 2 — Figure S1 [file 41419_2020_2487_MOESM2_ESM.tif]
